# Applying Hybrid ARIMA-SGARCH in Algorithmic Investment Strategies on S&P500 Index

**DOI:** 10.3390/e24020158

**Published:** 2022-01-20

**Authors:** Nguyen Vo, Robert Ślepaczuk

**Affiliations:** 1 Quantitative Finance Research Group, Faculty of Economic Sciences, University of Warsaw, Ul. Długa 44/50, 00-241 Warsaw, Poland; ainguyen.vo92@gmail.com; 2 Quantitative Finance Research Group, Department of Quantitative Finance, Faculty of Economic Sciences, University of Warsaw, Ul. Długa 44/50, 00-241 Warsaw, Poland

**Keywords:** algorithmic investment strategies, ARIMA, ARIMA-SGARCH, ARIMA-EGARCH, hybrid model, stock returns forecast, model robustness, sensitivity analysis

## Abstract

This research aims to compare the performance of ARIMA as a linear model with that of the combination of ARIMA and GARCH family models to forecast S&P500 log returns in order to construct algorithmic investment strategies on this index. We used the data collected from Yahoo Finance with daily frequency for the period from 1 January 2000 to 31 December 2019. By using a rolling window approach, we compared ARIMA with the hybrid models to examine whether hybrid ARIMA-SGARCH and ARIMA-EGARCH can really reflect the specific time-series characteristics and have better predictive power than the simple ARIMA model. In order to assess the precision and quality of these models in forecasting, we compared their equity lines, their forecasting error metrics (MAE, MAPE, RMSE, MAPE), and their performance metrics (annualized return compounded, annualized standard deviation, maximum drawdown, information ratio, and adjusted information ratio). The main contribution of this research is to show that the hybrid models outperform ARIMA and the benchmark (Buy&Hold strategy on S&P500 index) over the long term. These results are not sensitive to varying window sizes, the type of distribution, and the type of the GARCH model.

## 1. Introduction

Over the past few decades, time series forecasting in finance has been an interesting and important research area. It has attracted the attention of not only the researcher community but also investors, speculators, and governments who are interested in verification of various models and approaches in predicting of future prices of various types of assets (Sakowski and Turovtseva, 2020 [1], Torre-Torres et al. (2021) [2]) with the use of more standard tools (Goubuzaite and Teresiene, 2021 [3], Ivanyuk, 2021 [4]) or the newest ML techniques (Chlebus et al., 2021) [5] The main aim of time series modeling is to carefully measure and analyze the historical observations of the time series in order to develop the most appropriate models. The most important function of these models is to forecast future values of the time series, i.e., to predict the movements, behaviors, and changes, usually by reflecting the characteristics of the historical observations. It is obvious that to obtain adequately low forecasting errors, a proper process for model fitting needs to be taken into consideration. Along with the development of more advanced forecasting techniques, a lot of effort has been put into improving forecasting accuracy by choosing, testing, and fitting more efficient models. As a result, various important theories and assumptions about modeling forecast have evolved. In particular, the analyses of the time series of an essential US stock index, such as the S&P500 have never failed to get attention and efforts from those interested in quantitative finance.

One of the most popular and frequently used time-series models is the autoregressive integrated moving average (ARIMA). In this model, there is a linear relationship between past observation values (autoregressive) and random errors (moving average), in which random errors are assumed to be independent and identically distributed (i.i.d.) with a mean of zero and a constant variance $\sigma_{\varepsilon }^{2}$ over time. It is not so surprising that financial time series often do not follow this assumption, and the S&P500 index is no exception. Its returns can be extremely volatile during booms and busts. This means that the existence of volatility clustering in time series can affect the forecasting performance of the mean models like ARIMA. Therefore, most researchers have started to use symmetric generalized autoregressive conditional heteroscedasticity (SGARCH) and its family models when modeling volatility in order to obtain accurate forecasts. This is the reason why, in our study, we evaluate and compare the forecasting performance among the ARIMA model, the hybrid ARIMA-SGARCH and ARIMA-EGARCH (EGARCH-Exponential Generalized Autoregressive Conditional Heteroscedasticity is a modified version of GARCH).

In that context, the paper addresses one main hypothesis (RH): whether the ARIMA(p,1,q)-SGARCH(1,1) (hybrid model) with window size s = 1000 can generate a trading strategy that outperforms ARIMA(p,1,q). In other words, the key objective of this study is to verify the efficiency of forecasts from hybrid and simple ARIMA models in algorithmic trading strategies.

Based on this hypothesis, a few research questions are constructed:

RQ1. Is the result obtained from the main test robust to varying family of GARCH models?
RQ2. Does the hybrid model with EGARCH outperform the one with SGARCH?
RQ3. Is the result obtained from the main test robust to varying window sizes?
RQ4. Is the hybrid model sensitive to different window sizes?
RQ5. Is the result obtained from the main test robust to varying distributions?
RQ6. Is the hybrid model sensitive to different distributions?

In order to verify the main hypothesis and answer the research questions mentioned above, empirical research was conducted based on the dataset of the S&P500 index. This study includes several novelties in comparison to other papers focused on similar topics. The data were collected on a daily basis over the period from 1 January 2000 to 31 December 2019. Firstly, we conducted a rolling forecast based on the ARIMA model with window size s = 1000, while the most common approach and not necessarily the best one was based on the simple division of the whole data sample into one in-sample and one out-of-sample set (usually 70/80% to 30/20%). The optimized combination of p and q which had the lowest Akaike information criterion (AIC) was used to predict the return for the next day. For the purpose of the out-of-sample results, the vector of forecasted values had a length of 3530 elements (starting on 20 December 2005). Secondly, we describe and review our implementation of ARIMA(p,1,q)-SGARCH(1,1) models with generalized error distribution (GED) and window size equal to 1000, in which optimized ARIMA(p,1,q) is taken from the first step. Thirdly, we evaluate the performance of SGARCH with different window sizes as well as various distributions to check the sensitivity of the results obtained in the main test. EGARCH, known as another family of GARCH models, was also applied in the sensitivity analysis in order to check the robustness of our initial assumptions. Therefore, we propose a more thorough approach, checking various types of GARCH models and various assumptions concerning the error distributions. In order to examine the precision and the quality of these models in predicting and their efficiency in algorithmic investment strategies, we compare their equity lines, their error metrics (mean absolute error (MAE), mean absolute percentage error (MAPE), root mean squared error (RMSE)) and their performance metrics: annualized return compounded (ARC), annualized standard deviation (ASD), maximum drawdown (MD), information ratio (IR), and adjusted information ratio (AIR). Therefore, the precision of forecasts is verified in the two-step procedure which combines the evaluation of econometric model forecasts with standard error metrics and the evaluation of investment signals constructed on these forecasts with the help of performance metrics calculated based on final equity lines. We expect that hybrid models can help build more efficient algorithmic investment strategies which outperform ARIMA.

The paper is structured as follows: Section 2 provides a literature review and an overview of ARIMA, SGARCH, EGARCH, and the hybrid models. Section 3 presents all the details concerning the dataset and explains the methodology of ARIMA and the hybrid models. In addition, it gives a full description of trading strategy construction and the technique of generating buy/sell signals for the investment strategy. Section 4 discusses the empirical results and conducts some sensitivity analysis to determine whether the result is robust to initial assumptions. The Section 5 draws conclusions and makes suggestions for future works.

## 2. Literature Review

According to Strickland [6], there are various ways to classify methods for time series analysis. Some examples are frequency-domain and time-domain and parametric and non-parametric. The category used in this study is linear and non-linear regression. Regression analysis is a process of estimating the relationship between a dependent variable and one (or more than one) independent one(s). If there is only a single independent variable, this is known as simple linear regression; otherwise, it is called multiple regression. Both of these models assume that the dependent variable is continuous.

### 2.1. Linear Forecasting Models—Autoregressive Integrated Moving Average Models

Box and Jenkins [7] first introduced Autoregressive Integrated Moving Average Models (ARIMA) in 1976. This model describes the linear relationship between past observation values and random errors (also known as shocks or disturbances). In order to estimate the ARIMA model correctly, we must identify and remove non-stationarity through differencing; hence, the differences between a value and its lagged values ($y_{t}-Ly_{t})$ need to be calculated. The ARIMA model can be regarded as an extension of the ARMA model.

There are many related studies in modelling and forecasting stock prices using ARIMA models. For instance, Ariyo et al. [8] revealed an extensive process describing how to obtain the most appropriate ARIMA model to anticipate stock prices (based on the smallest value of Schwarz Information Criterion (SIC). Later, Kamruzzaman et al. [9] calculated returns by using the relative difference method and chose the ARIMA(2,1,2) model (based on the smallest value of AIC) as the most superior model for forecasting the stock market returns of the Dhaka Stock Exchange in Bangladesh. In addition, Abbasi et al. [10] applied this linear process into the flying cement industry and suggested that ARIMA(1,2,1) was a parsimonious model for forecasting cement stock prices in their case study.

### 2.2. Non-Linear Forecasting Models

#### 2.2.1. Autoregressive Conditional Heteroskedasticity—ARCH(q)

This model was first proposed by Engle [11] to predict the conditional variance of return series. Despite the key strength as a simple model which produces volatility estimates with positive excess kurtosis (It means fat tails relative to the normal distribution which is in line with empirical observations about returns.), its weaknesses should also be taken into consideration. Firstly, due to the possible large value of the lag q, it could lead to a large number of parameters to be estimated. Hence it may result in difficulties to determine parameters [12].

Secondly, as it is well known in practice, the stock prices or financial assets in general react differently to positive and negative shocks. However, ARCH models assume these kinds of shock have the same effects on the volatility as they depend on the square of the previous shocks. As a result, this weakness should be taken into consideration in forecasting when applying ARCH models [13]. Additionally, since ARCH models respond slowly to large shocks, they are likely to overpredict the volatility [14]. Furthermore, the ARCH models do not provide any new insights to understand the source of volatility of financial time series. They merely provide a mechanical way to describe the behavior of the conditional variance and give no indication of what causes such behavior to occur [15].

#### 2.2.2. Generalized Autoregressive Conditional Heteroscedasticity—GARCH(p,q)

The GARCH model is considered an extension of the ARCH model and was proposed by Bollerslev [16] by developing the symmetric GARCH (SGARCH). As demonstrated by many researchers and studies, the SGARCH (1,1) process is able to represent the majority of the time series [17]. The dataset which requires a model of higher orders like SGARCH(1,2) or SGARCH(2,1) is very rare [11]. However, financial time series inherits many characteristics that SGARCH is not able to incorporate well. Therefore, extensive generalizations with further features have been put forward in the literature.

One of the most essential properties of volatility that should be taken into consideration is the leverage effect, which describes the fact that there is a difference in reaction of volatility between notable price rises and notable price falls. This has led to the introduction of asymmetric GARCH models by purely adjusting the error term in the variance equation with a parameter to be responsible for this effect. These models were initially proposed by Engle et al. [18]. Nowadays, there are a variety of such models, including that by Higgins and Bera [19], in which a nonlinear asymmetric GARCH (N-GARCH) accounts for the leverage effect. Later, Glosten et al. [20] introduced the Glosten-Jagannathan-Runkle GARCH (GJR-GARCH) to precisely build up the volatility response from negative market shocks with an indicator function, while the quadratic ARCH (Q-ARCH) launched by Sentana [21] established asymmetric effects of both positive and negative shocks. The most recent model that allows for an asymmetric response due to the leverage effects is the exponential GARCH (EGARCH), which is discussed in the next Section.

#### 2.2.3. The Conditional Variance Equation: Exponential GARCH

The EGARCH, presented by Nelson [22], is supposed to avoid imposing constraints on the coefficients by specifying the logarithm of the conditional volatility. In reality, “bad news” typically has a larger impact on volatility than “good news”. In other words, by applying EGARCH, we can mitigate the disadvantage of using GARCH in which negative innovations tend to increase the volatility more than positive innovations with the same magnitude. EGARCH is applied in this paper in the section concerning the comparison of its performance with SGARCH’s.

#### 2.2.4. Underlying Return Distributions

GARCH models assume that the distribution of returns is normally distributed. However, this assumption has been proved in empirical financial markets to be inaccurate. This is because the distribution of financial returns tends to be leptokurtic [23]. It means that the tails are heavier in comparison with normal distribution. As a consequence, several fat tail distributions are applied in order to overcome this shortcoming. For instance, the Student-t was introduced by Bollerslev [24], the generalized error distribution (GED) by Nelson [22], and their skewed versions, which are all for leptokurtic distribution analysis.

### 2.3. The Hybrid ARIMA-GARCH

The class of ARIMA models with ARCH errors was proposed initially by Weiss [25]. The techniques were applied to U.S. macroeconomic time series. This approach was later adopted and extended by many researchers for modelling time series in various fields (e.g., Jabłecki et al. [26], Catellano and Ślepaczuk (2021) [27], Hauser and Kunst [28], Kijewski and Ślepaczuk [29]).

Alongside the theory development of the hybrid models in forecasting economic time series, Yaziz et al. [30] analyzed the performance of ARIMA-GARCH in forecasting gold price. The empirical results of 40-day gold price data series indicate that the hybrid ARIMA(1,1,1)-GARCH(0,2) model provides superior results and effectively improves evaluating and predicting precision in comparison to the linear models. Later, Sun [31] proposed the hybrid models to model and predict the equity returns for three US benchmark indices: Dow Transportation, S&P500, and VIX. The observed results suggested hybrid models are appropriate for anticipating the equity returns but have not been explored in the previous works. The latest work discussed in this Section is by Ismail and Mustapa [32]. They presented the assessment in building the best fitted ARIMA-GARCH model to generate predicting values of the S&P500 stock prices. The ARIMA(2,1,2)-GARCH(1,1) model was determined to be the most appropriate model for forecasting stock prices.

Collectively, the presented papers reveal that heteroskedasticity can affect the validity or power of statistical tests when using ARIMA models, and the ARCH effect should be considered. Furthermore, the mentioned studies also indicate that the combination of ARIMA and the family of GARCH should be expected to perform well in modelling financial time series. In our study, we fit an optimal ARIMA-SGARCH as well as ARIMA-EGARCH in different window sizes and various return distributions in algorithmic investment strategies on the S&P500 index. It is based on the AIC, each day, using the rolling window approach.

## 3. Methodology and Data

In this Section, we provide a complete data analysis and a model-fitting procedure for the logarithmic returns of the S&P500 index. Additionally, this Section covers the description of methodology.

### 3.1. Data Analysis

#### 3.1.1. Data Fetching and Preprocessing of Historical Data

We fetched the historical data from Yahoo Finance. Given the hybrid ARIMA-GARCH model proposed in Section 1, the data for the S&P500 index were collected with the period of 19 years for sufficiently reliable model fitting and forecasting purposes.

The first step in the process was cleaning the data. Then, we transformed the adjusted price into a daily logarithmic return, which was calculated according to the following formula:

(1) $$r_{t}=\ln\left(\frac{P_{t}}{P_{t-1}}\right)$$

There are many reasons for choosing log returns instead of normal prices as a variable to forecast in this study. First, they can be added across time periods in order to create cumulative returns. Second, it is easy to convert between log return and simple return. Last but not least, log return follows normal distribution. Why is it an important advantage? This kind of distribution is solely dependent on the mean and the standard deviation of the sample. Based on these characteristics, any variable that exhibits normal distribution is feasible to be forecasted with higher accuracy, and our variable, in this case, is log return. Moreover, stock prices cannot be modelled by normal distribution because they have a negative side, and stock prices cannot fall below zero. In other words, prices are log-normally distributed, then the logarithm of each price will have a normal distribution. These relationships can be expressed by the equation below:

(2) $$\ln\left(\frac{P_{t}}{P_{0}}\right)=\ln(P_{t)}-\ln(P_{0)}=\ln\left(1+r_{t}\right)$$

#### 3.1.2. Descriptive Statistics

Table 1 presents the descriptive statistics of the adjusted closing prices and log returns of the S&P500 index for the whole dataset.

**Figure 1:**
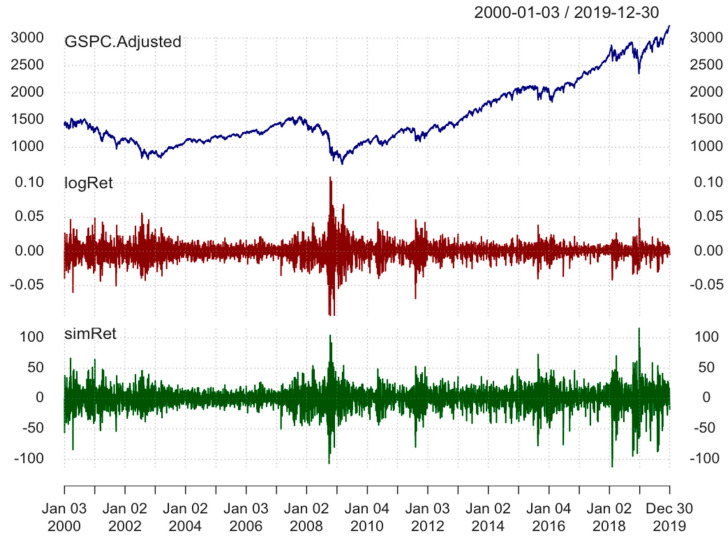
S&P500 index prices with its first differences and log returns. The fluctuations of S&P500 index prices, its first differences and log returns of S&P500 index prices in the period between 1 January 2000 and 31 December 2019.

As can be seen from Figure 1, there are a few periods, such as 2008, 2011, 2015, and 2018, that show high volatility of returns. Therefore, we can expect to build more accurate forecasting models if we are able to mitigate and “smooth” such periods. This is further explained in the Methodology section.

Next, when we consider the central tendency, Table 1 shows two types of estimation as mean and median. The central tendency of a distribution is an estimation of the “center” of distribution, in this case of stock prices and log returns. If mean (or average) is computed by added up all the values and divided by the number of values, median, on the other hand, is the middle value or midpoint in data (also known as 50th percentile). In a normal distribution, these two metrics fall at the same midline point. In other words, mean and median are equal. In this study, with mean of 1574.6801 and median of 1360.9550, our initial assessment about stock prices of S&P500 is that they are not normally distributed. This it makes sense since normal distribution has 2 sides, while stock prices cannot be negative (below zero).

**Figure 2:**
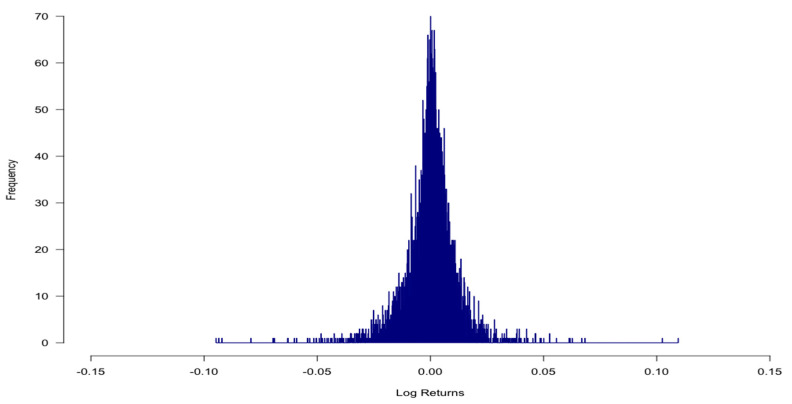
Histogram for log returns–S&P500. The histogram of S&P500 index log returns in the period between 1 January 2000 and 31 December 2019.

Figure 2 shows that log return series, on the other hand, has different characteristics of distribution in comparison to stock prices dataset. Intuitively, log returns are normally distributed because mean and median values are close to each other (0.0002 and 0.0005, respectively). Furthermore, values of first and third quantile (−0.0047 and 0.0057, respectively) as well as min and max values (−0.0947 and 0.1096, respectively) are quite symmetric. It is the main reason why we use log returns to build models.

**Figure 3:**
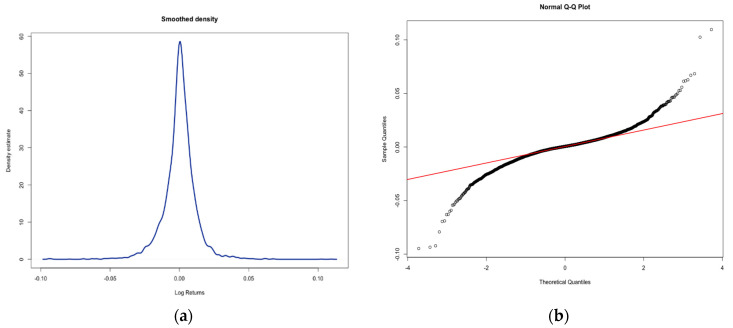
Smoothed density and Q-Q plot for log returns–S&P500. Smoothed density (**a**) and Q-Q plot (**b**) for S&P500 index log returns in the period between 1 January 2000 and 31 December 2019.

However, the kurtosis value of 8.6448 is larger than 3 (hence it is named leptokurtic) and skew value of −0.2295 (near but below zero), so we could say the log returns series is similar to double exponential distribution (https://www.itl.nist.gov/div898/handbook/eda/section3/eda35b.htm accessed on 15 July 2021). This kind of distribution is symmetric, but compared to the normal one, it has a stronger (higher and sharper) peak, more rapid decay, and heavier tails. Furthermore, looking at the histograms in Figure 3, it is perfectly clear that they show certain similarities to the normal distribution.

Finally, when taking dispersion into consideration, if standard error (SE) of S&P500 prices is far away from zero (8.2559), log return series’ one, on the other hand, is quite close to zero (0.0002). SE can be approximated by the following formula:

(3) $$SE=\frac{\sigma }{\sqrt{n}}$$

where: *σ* is standard deviation and *n* is the number of observations (sample size).

Equation (3) informs us that the larger the sample size (more data points) involved in the calculation, the smaller the SE tends to be. That is, if SE is small, the data are said to be more representative of the true mean. So, with the value of 8.2559, SE of S&P500 prices shows that data may have some notable irregularities as the sample is less accurate (due to high value of SE). Obviously, with SE of 0.0002 (≈0), log return dataset can be expected to build more accurate models.

### 3.2. Methodology

#### 3.2.1. Fundamental Concepts and Definitions

##### Autoregressive Moving Average Models—ARMA(p,q)

The ARMA process is the combination of the autoregressive model and moving average [2] designed for a stationary time series. Autoregression (AR) describes a stochastic process, and AR(p) can be denoted as shown below:

$$\mathrm{AR}(1)\;:\;y_{t}=\phi y_{t-1}+\varepsilon_{t}$$

(4) $$\mathrm{AR}(p)\;:\;y_{t}=\phi_{1}y_{t-1}+\phi_{2}y_{t-2}+\ldots +\phi_{p}y_{t-p}+\varepsilon_{t}\;$$

where: $\phi_{p}$ denotes the weights given to past observations at each lag, *p* is a positive integer providing the number of lags to be included, and $\varepsilon_{t}$ is white noise.

We now introduce the lag, i.e., in the interest of notational convenience, which simply produces the previous element of the series, as shown below:

(5) $$Ly_{t}=y_{t-1}\;$$

So, an AR (p), using lag notation, is now:

(6) $$\left(1-\overset{p}{\underset{i=1}{\sum }}\phi_{i}L^{i}\right)y_{t}=c+\varepsilon_{t}\;\;\;,p=1,2,\ldots$$

The moving average process of order q is denoted as MA(q) and the created time series contains a mean of q lagged white noise variables shifting along the series.

(7) $$\mathrm{MA}(1)\;:\;y_{t}=\mu +\varepsilon_{t}+\theta_{1}\varepsilon_{t-1}\;\mathrm{MA}(q)\;:y_{t}=\mu +\varepsilon_{t}+\theta_{1}\varepsilon_{t-1}+\ldots +\theta_{q}\varepsilon_{t-q}\;$$

where: μ is the mean of the series, and $\theta_{q}$ are the weights given to each white noise value. Using lag notation, MA(q) can be written:

(8) $$y_{t}=\mu +\left(1+\overset{q}{\underset{i=1}{\sum }}\theta_{i}L^{i}\right)\varepsilon_{t},\;\;\;\;\;q=1,2,\ldots \;$$

ARMA(p,q) is now expressed as below:

(9) $$y_{t}=\phi_{1}y_{t-1}+\phi_{2}y_{t-2}+\ldots +\phi_{p}y_{t-p}+\varepsilon_{t}-\theta_{1}\varepsilon_{t-1}+\ldots -\theta_{q}\varepsilon_{t-q}\;$$

where $\varepsilon_{t}$ is independent of $y_{t-1},\;y_{t-2}$, …

##### Autoregressive Integrated Moving Average Models—ARIMA(p,d,q)

The ARIMA model can be regarded as an extension of the ARMA model [7]. This process can be written as:

(10) $$\left(1-\overset{p}{\underset{i=1}{\sum }}\phi_{i}L^{i}\right){\left(1-L\right)}^{d}y_{t}=c+\left(1+\overset{q}{\underset{i=1}{\sum }}\theta_{i}L^{i}\right)\varepsilon_{t}\;$$

where:

d is the number of differencing done to the series to achieve stationarity (Shumway and Stoffer [33])with I (d):

(11) $${\left(1-L\right)}^{d}y_{t}=\mu +\varepsilon_{t}\;$$

p is the number of autoregressive terms (AR)

q is the number of moving average terms (MA)

##### The Autoregressive Conditional Heteroskedasticity—ARCH(q)

ARCH model [6] can be expressed as:

(12) $$y_{t}=C+\varepsilon_{t}\;,\varepsilon_{t}=z_{t}\sigma_{t}\;$$

where:

$y_{t}$ is an observed data series

C is a constant value

$\varepsilon_{t}$ is residual

$z_{t}$ is the standardized residual, independently and identically distributed with mean equal to 0 and variance tends toward 1 as sample size tends toward infinity, $\sigma_{t}$ is the square root of the conditional variance, and it is a non-negative process.

ARCH(q) can be expressed in the following equation:

(13) $$\sigma^{2}{}_{t}=\alpha_{0}+\overset{q}{\underset{i=1}{\sum }}\alpha_{i}r^{2}{}_{t-i}\;$$

with $\alpha_{0},$ $\alpha_{i}\;\geq \;0\;\left(i=1,\;\ldots \;,\;q\right)\;so\;\sigma^{2}{}_{t}$ is non-negative.

##### Generalized Autoregressive Conditional Heteroscedasticity—GARCH(p,q)

The GARCH model is considered to be an extension of an ARCH model [16]. Unlike ARCH which involves only the most recent returns, generalized ARCH (GARCH) enhances the accuracy of forecasting by adding all the past squared returns with higher weights on more recent data and lower ones for faraway lags. Furthermore, GARCH is more restrained in comparison to ARCH, hence it can avoid overfitting and permit an infinite number of past squared errors to impact the current conditional variance [34]. So now, the conditional variance $\sigma^{2}$ is expressed by GARCH(p,q) as:

(14) $$\sigma^{2}{}_{t}=\alpha_{0}+\overset{q}{\underset{i=1}{\sum }}\alpha_{i}r^{2}{}_{t-i}+\overset{p}{\underset{j=1}{\sum }}\beta_{j}\sigma^{2}{}_{t-j}\;$$

GARCH(1,1) can be expressed by the equation below:

(15) $$\sigma^{2}{}_{t}=\alpha_{0}+\alpha_{1}r^{2}{}_{t-1}+\beta_{1}\sigma^{2}{}_{t-1}\;\;\;\;with\;0\;<\alpha_{1}\;+\;\beta_{1}\;<\;1$$

and the rate of decay governed by $\alpha_{1}$ + $\beta_{1}$ where the closer $\alpha_{1}$ + $\beta_{1}$ is to 1, the slower the decay of the autocorrelation is. As proven by Bollerslev et al. [35], the valuations of GARCH(1,1) for stock returns usually yield $\alpha_{1}$ + $\beta_{1}$ very close to 1.

##### The Conditional Variance Equation: Exponential GARCH

The EGARCH model [17] is defined as $a_{t}=e_{t}\sigma_{t}$, in which:

(16) $$ln(\sigma^{2}{}_{t})\;=\;\alpha_{0}+g\left(e_{t-1}\right)+\beta_{1}ln\left(\sigma^{2}{}_{t-1}\right)\;$$

The function $g\left(e_{t-1}\right)$ determines the asymmetry and is defined as the weighted innovation:

(17) $$g\left(e_{t-1}\right)=\alpha_{1}e_{t-1}\;+\;\gamma_{1\;}\left[\left|e_{t-1}\right|-E\left(\left|e_{t-1}\right|\right)\right]\;$$

where: $\alpha_{1}\;$ and $\gamma_{1\;}$ are real constants. This means the model can be written:

(18) $$ln(\sigma^{2}{}_{t})\;=\;\alpha_{0}+\alpha_{1}e_{t-1}\;+\;\gamma_{1\;}\left[\left|e_{t-1}\right|-E\left(\left|e_{t-1}\right|\right)\right]+\beta_{1}ln\left(\sigma^{2}{}_{t-1}\right)\;$$

Equation (18) informs us that a positive shock has the effect $(\alpha_{1}+$ $\gamma_{1\;})e_{t-1}$, while the effect of the negative one has $(\alpha_{1}-$ $\gamma_{1\;})e_{t-1}$. In reality, “good news” typically exerts a smaller impact on the volatility than “bad news”. For this reason, the use of $g\left(e_{t-1}\right)$ allows the model to respond asymmetrically to “new information” in the market.

Equation (19) expresses the general EGARCH(s,r) models.

(19) $$ln(\sigma^{2}{}_{t})=\alpha_{0}+\overset{s}{\underset{i=1}{\sum }}g_{i}e_{t-i}+\overset{r}{\underset{j=1}{\sum }}\beta_{j}ln\left(\sigma^{2}{}_{t-j}\right)\;$$

##### The Hybrid ARIMA-GARCH

As discussed above, ARIMA models are proposed for stationary time series with the assumption of constant variance, defined as “homoskedasticity”, while financial time series data often do not follow these assumptions. In practice, stock prices can be tremendously volatile during economic growth as well as recessions. In such scenarios, when homoskedasticity presumption is violated, it is said that the errors are heteroskedastic (a phenomenon known as heteroskedasticity). In other words, since heteroskedasticity is present, ARIMA or linear regression in general gives equal weights to all observations when observations with larger disturbance variance contain less information than the ones with smaller disturbance variance [36]. Given that heteroskedasticity can affect the validity or power of statistical tests when using ARIMA models, the ARCH effect should be considered.

Furthermore, according to Mandelbrot [37], large changes tend to be followed by large changes and vice versa. If volatility of a series exhibits such characteristics, it suggests that past variances might be predictive of the current variance. Hence, ARCH and GARCH models are the appropriate options in not only capturing the variance of each error term and correcting the deficiencies of heteroskedasticity for least squares but also dealing with the issue of volatility clustering. If one mechanism can simultaneously predict both the conditional mean and the conditional heteroscedasticity of the process, it is suggested as hybrid ARIMA-GARCH. It combines an ARIMA specification for modelling the mean behavior with the family of GARCH functions for simulating, estimating, and forecasting the variance behavior of the residuals from the ARIMA model. The hybrid ARIMA(p,d,q)-GARCH(r,s) can be specified as:

(20) $$\begin{array}{c} y_{t}=\phi_{1}y_{t-1}+\phi_{2}y_{t-2}+\ldots +\phi_{p}y_{t-p}+\varepsilon_{t}-\theta_{1}\varepsilon_{t-1}+\ldots -\theta_{q}\varepsilon_{t-q}\\ \sigma^{2}{}_{t}=\alpha_{0}+\overset{q}{\underset{i=1}{\sum }}\alpha_{i}r^{2}{}_{t-i}+\overset{p}{\underset{j=1}{\sum }}\beta_{j}\sigma^{2}{}_{t-j}\\ \varepsilon_{t}=z_{t}\sigma_{t}\;(\varepsilon_{t}:N(0,\;\sigma^{2}{}_{t})) \end{array}$$

#### 3.2.2. Overview of the Methodology and Input Parameters

In order to achieve the goals that were mentioned in the Introduction section, the methodology of this research is structured in the following way:

Firstly, we conducted a rolling forecast based on an ARIMA model with window size(s) equal to 1000. The optimized combination of p and q which has the lowest AIC is used to predict return for the next point. At the end, the vector of forecasted values has the length of 3530 elements, with a starting point at 20 December 2005.

Next, we describe and review our implementation of dynamic ARIMA(p,1,q)-SGARCH(1,1) models with GED distribution and window size(s) equal to 1000 and where optimized ARIMA(p,1,q) is taken from the first step. Then, we evaluate the results based on error metrics, performance metrics, and equity curves.

After that, in the sensitivity analysis section, we build hybrid models with different input parameters: window size(s) equal to 500 and then 1500, with the following distributions: SNORM, SSTD, SGED.

Finally, we replaced SGARCH by EGARCH. We also conduct forecasting ARIMA on different window sizes in order to have a final conclusion regarding whether the hybrid model outperforms ARIMA in different input variables. We used the same criteria as in the main test to compare and evaluate the performance of each model.

To sum up, the forecasting models are centered around five sets displayed in Table 2 below.

#### 3.2.3. The Implementation of Forecasting Models

##### ARIMA(p,1,q)

**Figure 4:**
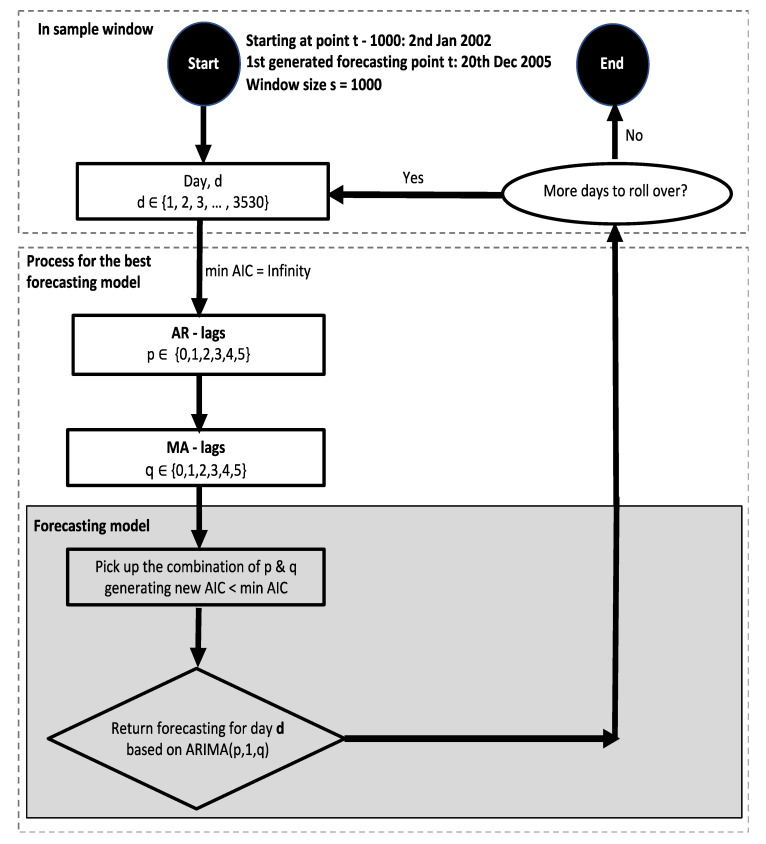
Flowchart of the forecasting model ARIMA(p,1,q). This flowchart is for models with window size s = 1000. For various values of window size, such as 500 or 1500, the first generated forecasting point t is the same, hence all models have the same length of forecasted log return vector of 3530. The difference here is the starting point where models select the first point to start a training model. For s = 500, the starting point should be at t − 500, and similarly, for s = 1500, the starting point should be at t − 1500.

This section gives an in-depth outline of the actual implementation of ARIMA(p,1,q). As can be seen from the flowchart in Figure 4, this process spans fitting and forecasting from selecting sample size until the one-day-ahead return is obtained. Additionally, the model assessment framework will be provided, including return generating properties and an overview of the model’s computational complexity.

**Figure 5:**
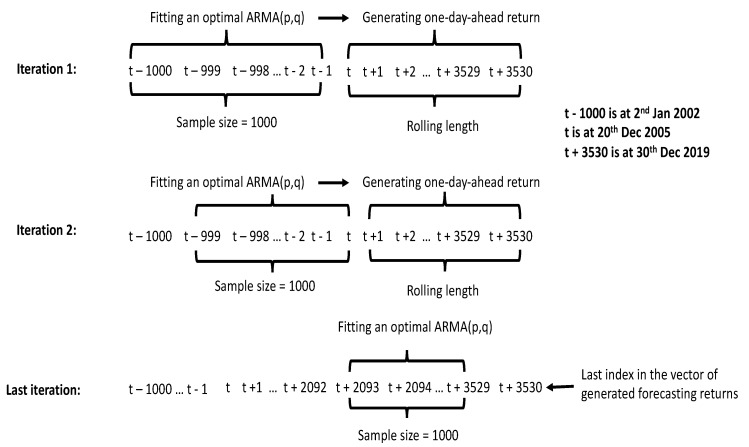
Rolling window illustration for sample size s = 1000. This illustration is for models with window size s = 1000 and applied the same for both the ARIMA and hybrid models in the next section. For various values of window size, such as 500 or 1500, the process is the same except the sample size understanding as window length. For s = 500, the starting point should be at t − 500, and similarly, for s = 1500, the starting point should be at t − 1500.

One optimal ARIMA(p,1,q) forecasting model is fitted using a rolling window approach with different combinations of p and q for the values of the input variables. This optimized model which has the lowest value of AIC is used to generate one-day-ahead return. Since the rolling window approach is applied, the next data point is estimated based on the sample size equal to the length of window. The mechanism of this method is illustrated more specifically in Figure 5, with three iterations and sample size(s) equal to 1000. With the starting point at 20 December 2005, we have 3530 forecasted values for the ARIMA model, based on which we have constructed equity lines for each strategy.

Testing one combination of p and q is referred to as one iteration. An important condition we set up in this loop is that p and q cannot be equal to 0 at the same time. This means that ARIMA(0,1,0) is excluded. The best fitting model is selected based on the lowest AIC. In each iteration, the inner loop compares 6 ∗ 6 − 1 = 35 models together to pick up the best one with the lowest value of AIC. To put it in another way, excluding the situation p = q = 0, with 6 values from 0 to 5, p and q generate 35 combinations. Hence, with the starting point t at 20 December 2005, each point in the vector of 3530 elements is forecasted based on the most optimized ARIMA within these 35 models. For the whole process, the loop generates a predicting returns vector by checking 3530 ∗ 35 = 123,550 models in total.

#### 3.2.4. Dynamic ARIMA(p,1,q)-SGARCH(1,1)

**Figure 6:**
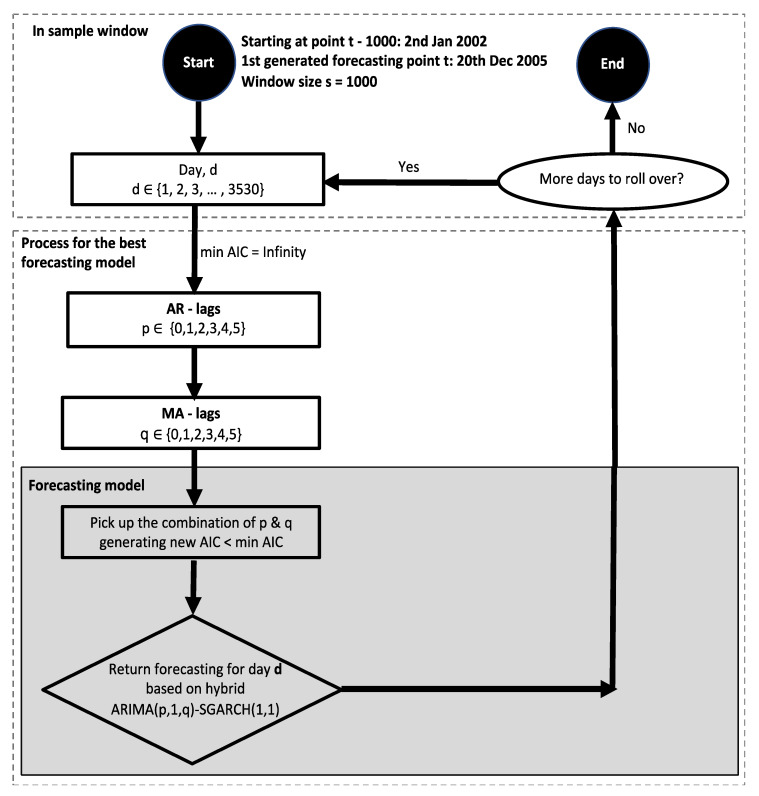
Flowchart of the forecasting model ARIMA(p,1,q)-SGARCH(1,1). This flowchart is for models with window size s = 1000. For various values of window size, such as 500 or 1500, the first generated forecasting point t is the same, hence all models have the same length of forecasted log return vector of 3530. The difference here is the starting point where models select the first point to start the training model. For s = 500, the starting point should be at t − 500 and similarly, for s = 1500, the starting point should be at t − 1500.

In this section, we describe and review the implementation of dynamic ARIMA(p,1,q)-SGARCH(1,1). The steps applied to select parameters are similar to those used to fit ARIMA models, and they are described in Figure 6. It has similar steps as mentioned in Figure 4 regarding optimizing input parameters to fit model as well as the rolling process in Figure 5. The return distributions GED and SGARCH(1,1) with window size s = 1000 are used in building hybrid models. We have ARIMA(p,1,q)-SGARCH(1,1) as an optimal outcome per iteration to forecast the next value of log return, where SGARCH is applied to model the nonlinear patterns of the residuals. In other words, the error term $\varepsilon_{t}$ of the ARIMA model in this process follows SGARCH(1,1) instead of being assumed constant like the ARIMA process in Figure 4.

#### 3.2.5. Trading Strategy Criteria

In general, the rule for going long (buy) or short (sell) is as follows: if the forecasted log return is positive at time t + 1, we go long (buy stocks) at time t (direction would be +1); if the forecasted log return is negative at time t + 1, we go short (sell stocks) at time t (direction would be −1); and if the forecasted direction at time t+1 is the same as at time t, then there are no changes.

The initial investment is assumed to be $1259.92 at the beginning. It is also the adjusted closing price of the S&P500 on 19 December 2005 (at t − 1), which is used as the starting point of the equity curves. For the benchmark, we used the Buy&Hold strategy on the S&P500 index in the period 20 December 2005–31 December 2019 and compared our strategy’s performance with this benchmark.

#### 3.2.6. Criteria and Evaluation of Statistic Fit and Forecasting

##### Akaike Information Criterion (AIC)

When a statistical model is selected to represent the process that generates the data, it will not be completely accurate. In other words, some “information” will be lost by applying this model in forecasting, and it might lead astray if the missing information is of great importance and has a huge effect on adopted data. However, there is a trade-off between the goodness of fit (how well the model fits a set of observations) and the number of parameters (more parameters -> more information) in the model. In order to avoid the risks of overfitting and underfitting, we apply the Akaike information criterion–AIC (https://www.statisticshowto.com/akaikes-information-criterion/, accessed on 15 July 2021). In general terms, AIC is an estimator of the relative quality of statistical models for a given dataset and also provides means for the model selection, which is expressed by the following formula:

(21) $$AIC=\;2k\;-\;2ln\;(\hat{L})$$

where: *k* is the number of estimated parameters in the model and $\hat{L}$ is the maximum value of the likelihood function for the model.

##### Error Metrics

In order to evaluate forecast form estimated models, we calculated the following error metrics:

mean absolute error (MAE)

(22) $$MAE=\frac{1}{n}\overset{n}{\underset{t=1}{\sum }}\left|A_{i}-F_{i}\right|\;$$

where: n is the number of errors; $A_{i}$ is the actual value and $F_{i}$ is the forecasted value computed by the given model.

mean square error (MSE)

(23) $$MSE=\frac{1}{n}\overset{n}{\underset{t=1}{\sum }}{\left(A_{i}-F_{i}\right)}^{2}\;$$

root mean square error (RMSE)

(24) $$RMSE=\sqrt{\frac{1}{n}\overset{n}{\underset{i=1}{\sum }}{\left(A_{i}-F_{i}\right)}^{2}}\;$$

mean absolute percentage error (MAPE)

(25) $$MAPE=\frac{1}{n}\overset{n}{\underset{t=1}{\sum }}\left|\frac{A_{i}-F_{i}}{A_{i}}\right|\;$$

##### Performance Statistics

Moreover, in order to evaluate the efficiency of algorithmic investment strategies built based on the signals from econometric models, we calculated the performance metrics based on the created equity lines and formulas from Kość et al. [38] and Zenkova and Ślepaczuk [39].

Annualized return compounded (ARC)

ARC is expressed as percentage (%) and computed as:

(26) $$ARC=\overset{N}{\underset{i=1}{\prod }}{\left(1+R_{i}\right)}^{252/N}\;–\;1\;$$

where: $R_{i}$ is the percentage rate of return and N is the sample size

Annualized standard deviation (ASD)

ASD is expressed as percentage (%) and computed as:

(27) $$ASD=\sqrt{252}\;*\sqrt{\frac{1}{N-1}\overset{N}{\underset{i=1}{\sum }}{\left(R_{i}-\bar{R}\right)}^{2}}$$

where: $R_{i}$ is the percentage rate of return, $\bar{R}$ is the average rate of return, and N is the sample size

Maximum drawdown (MD)

MD is the difference between the global maximum and the consecutive global minimum of the equity curve. The importance here is the time order, which means the global maximum must occur before the global minimum. It is expressed as below:

(28) $$MD\;{\left(S\right)}_{t_{1}}^{t_{2}}=max_{\left(x,y\right)\;\in \;\{{\left[t_{1},t_{2}\right]}^{2}\;:\;x\;\leq \;y\}}\frac{S_{x}-S_{y}}{S_{x}}\;$$

where: S is the price process; $\left[t_{1},t_{2}\right]$ is the period between time $t_{1}\;and\;t_{2}$

Information ratio (IR)

IR is the ratio between ARC and ASD informing us about risk adjusted returns for tested strategy

(29) $$IR=\frac{ARC}{ASD}\;$$

Adjusted information ratio (IR*)

IR* is similar to IR, but it also takes into account MD as one of the risk factors. Then we have:

(30) $$IR*=\frac{{\mathrm{ARC}}^{2}\;*\;\mathrm{sign}\left\{\mathrm{ARC}\right\}}{\mathrm{ASD}\;*\;\mathrm{MD}}$$

where: sign {ARC} is the sign of ARC and can take values of 0, −1 or +1.

## 4. Results and Robustness Tests

### 4.1. Results

The performance of ARIMA (ARIMA 1000) and hybrid model ARIMA(p,1,q)-SGARCH(1,1) with GED distribution (SGARCH.GED 1000) as well as benchmark (Buy&Hold–S&P500) for window size(s) equal to 1000 are presented in Table 3. As the result shows, the hybrid model outperforms ARIMA and benchmark strategy evaluated based on error metrics and performance statistics. These results are much better even if compared with the ensemble models built from ML techniques (LSTM model) for S&P500 index (Michańków et al.) [40] or rather complex approach using pair trading strategies for the constituents of the Nasdaq 100 index (Bui and Ślepaczuk) [41]. In particular, SGARCH.GED 1000 is more accurate than ARIMA 1000 in predicting returns and has the lowest values of MAE, MSE, RMSE, and MAPE (11.831 against 12.122; 303.044 against 310.372; 17.408 against 17.617; 0.00754 against 0.00775, respectively).

Concerning performance statistics, the hybrid model generates the highest IR among the 3 methods with 0.742, the second one is ARIMA 1000 with IR of 0.428, and the last one is the benchmark with IR of 0.368. Although the hybrid model gives the highest ARC equal to 14.026%, its ASD is also the highest with 18.893%. However, it is not significantly different from the lowest value of 18.826% belonging to the benchmark. In terms of adjusted IR*, we can see that SGARCH.GED 1000 also outperforms ARIMA 1000. The difference between IR and IR* is that we additionally take into account MD as a measure of risk beside ASD. We can see that MD of ARIMA (50.007%) is almost 2 times higher than the MD of the hybrid model (25.884%), while the ARC in the numerator of IR* (8.084%) is nearly 1.75 times lower than that of the hybrid model. As a result, the IR* of ARIMA 1000 is approximately 6 times smaller than that of SGARCH.GED 1000 (0.069 against 0.402). With this value of IR*, the hybrid method again beats the market with IR* of 0.045 (approximately 10 times higher).

**Figure 7:**
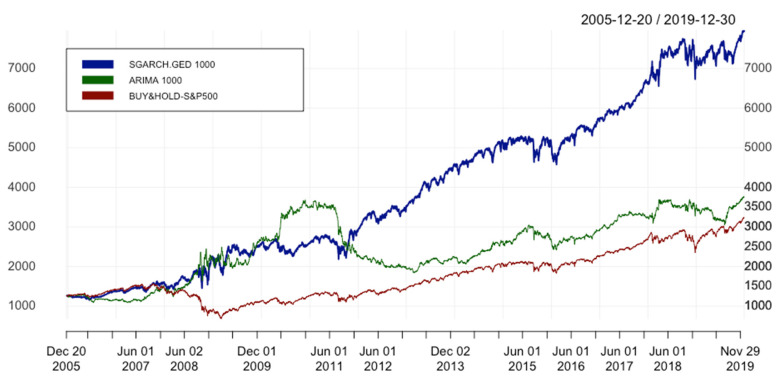
Equity curves of ARIMA(p,1,q) & ARIMA(p,1,q)-SGARCH(1,1). In order to simplify the structure of the legend, SGARCH.GED 1000 is understood as ARIMA(p,1,q)-SGARCH(1,1) with GED distribution and window size equal to 1000 days; ARIMA 1000 is ARIMA(p,1,q) with window size s = 1000; BUY&HOLD-S&P500 is the benchmark strategy.

To visualize the performance of ARIMA(p,1,q) and the hybrid model with GED distribution as well as the benchmark, the cumulative returns of these strategies are shown in Figure 7. The equity curves of ARIMA and the hybrid model remain below the Buy&Hold strategy for almost 2 years, but during the financial market crisis of 2008–2009, they behave tremendously well. In particular, from the latter half of 2010, ARIMA proved to be a good candidate, which even outperformed the hybrid model impressively in almost 2 years (2010–2011). However, at the end of 2011, ARIMA performance showed a dramatic decline and then remained below the hybrid model until the end of our data period. After the financial market crisis of 2008–2009, the hybrid model underwent an upward trend with small breaks until the end of 2019. In general, in spite of being under the ARIMA in short periods of time from 2008–2011, it is depicted as the most superior model in the whole discussed data frame. At the onset, it is clear it captures well all the movements of time series and is much better when compared with the benchmark.

The consequences of such results are very important from the point of view of the possibility of creating market-beating investment strategies and the allocation of assets by financial institutions actively managing assets on financial markets. Our results contradict the accepted Efficient Market Hypothesis in the informational sense in the weak form and should be the reason for further intensive research on this topic.

In general, error metrics, performance statistics, and equity curves imply that the hybrid model outperforms ARIMA and the benchmark. Referring to the main hypothesis of this paper, we can conclude that the combination between ARIMA(p,1,q) and SGARCH(1,1) is efficient.

### 4.2. Robustness Test

In this Section, we verify whether the results we obtained above are robust to varying family of GARCH, various distributions, as well as different window lengths. In the previously obtained results, we conducted rolling forecasting on a hybrid model ARIMA(p,1,q)-SGARCH with GED distribution and window size equal to 1000. In order to check the sensitivity of this result, we changed input parameters to conduct three extra tests. In particular, the first robustness test is to substitute SGARCH to EGARCH (keeping the same GED distribution and window size of 1000 days used in the main test). The second one is changing GED to a variety of distributions, such as SNROM, SSTD, and SGED (the other conditions of the main test remain unchanged). The last one is replacing the window size of 1000 to 500 and 1500 (the remaining conditions of the main test are kept the same).

#### 4.2.1. Varying Family of GARCH Models

Table 4 informs us that ARIMA(p,1,q) has the worst performance, with the highest values of MAE, MSE, RMSE, and MAPE in comparison to EGARCH.GED 1000 (12.122 against 11.828, 310.372 against 301.745, 17.617 against 17.371, 0.00775 against 0.00753, respectively). Moreover, with the highest values of all key performance indicators (KPIs), where ARC = 11.010%, IR = 0.582 and IR* = 0.220, EGARCH.GED 1000 beats not only ARIMA with ARC = 8.084%, IR = 0.428 and IR* = 0.069, but also the benchmark with ARC = 6.931%, IR = 0.368 and IR* = 0.045.

**Figure 8:**
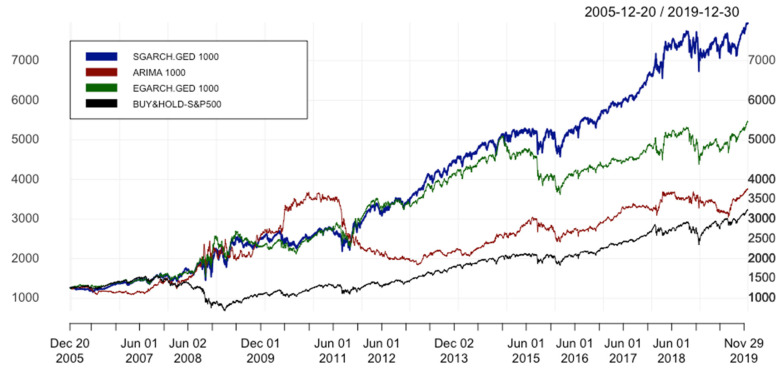
Equity curves of ARIMA(p,1,q) & ARIMA(p,1,q)-EGARCH(1,1). In order to simplify the structure of the legend, SGARCH.GED 1000 is understood as ARIMA(p,1,q)-SGARCH(1,1) with GED distribution and window size equal to 1000 days; EGARCH.GED 1000 is understood as ARIMA(p,1,q)-EGARCH(1,1) with GED distribution and window size equal to 1000; ARIMA 1000 is ARIMA(p,1,q) with window size s = 1000; BUY&HOLD-S&P500 is the benchmark strategy.

The equity curves of all models and the benchmark are plotted in Figure 8. In general, despite being under ARIMA in some periods of time from 2008–2011, EGARCH.GED 1000 is depicted as a superior model in the whole analyzed data period. It leads to the conclusion that the transformation from SGARCH to EGARCH seems to be insensitive. That is to say, we can conclude that the combination of ARIMA(p,1,q) and EGARCH(1,1) outperforms ARIMA in a similar way as the combination of ARIMA(p,1,q) and SGARCH(1,1), and we can treat it as the answer to the first research question of this paper.

However, as can be seen in Table 4, although error metrics of EGARCH.GED.1000 have the lowest values, it cannot beat the SGARCH.GED 1000 in terms of performance statistics. With the ARC of 14.026%, IR* of 0.402, the hybrid model with SGARCH is the most superior strategy in comparison with the other 3 methods in Table 4. Furthermore, Figure 8 shows that from the beginning of 2016, we observe a big difference in the cumulative returns of these two hybrid models. EGARCH is introduced as more advanced than SGARCH since it takes the magnitude of volatility into consideration. In other words, EGARCH mitigates the disadvantage of GARCH by putting more weight on negative innovation since it tends to increase the volatility. However, the result based on IR*, which is selected as the most important performance statistic to evaluate the model, does not support this theory. This leads to the observation that the best model is not necessarily the same when the selection is based on the best error metrics or the best performance statistics. In a nutshell, as a response to the second research question in this paper, based on IR* as the main indicator for selecting the best model, SGARCH.GED 1000 outperforms EGARCH.GED 1000.

#### 4.2.2. Varying Window Sizes

Table 5 demonstrates the performance of ARIMA and SGARCH.GED with window sizes of 500 and then 1500. The result of the main test with window size = 1000 is also included. Error metrics for the window size of 500 show that SGARCH.GED 500, with the lower values of MAE, MSE, RMSE, and MAPE (11.91 against 12.216, 307.812 against 318.342, 17.545 against 17.842, 0.00758 against 0.00777, respectively), outperforms ARIMA 500. Moreover, with the higher values of KPIs in performance statistics (ARC = 5.912%, IR = 0.313 and IR* = 0.052), SGARCH.GED 500 also beats ARIMA 500 (ARC = −0.574%, IR = −0.03 and almost zero for IR*).

When window size is switched from 500 to 1500, we have the same results in which the hybrid models are demonstrated to be superior to ARIMA. Particularly, SGARCH.GED 1500 has an ARC of 12.186%, which is almost 2.5 times higher than that of ARIMA (5.005%), and ARIMA’s IR* = 0.026 is nearly 10 times lower than that of the hybrid models (0.304). The difference in the result of IR* is because of the lower value of ARC as the numerator and the higher value of MD as the denominator leading to the lower final value of ARIMA’s IR* in comparison with the hybrid models.

**Figure 9:**
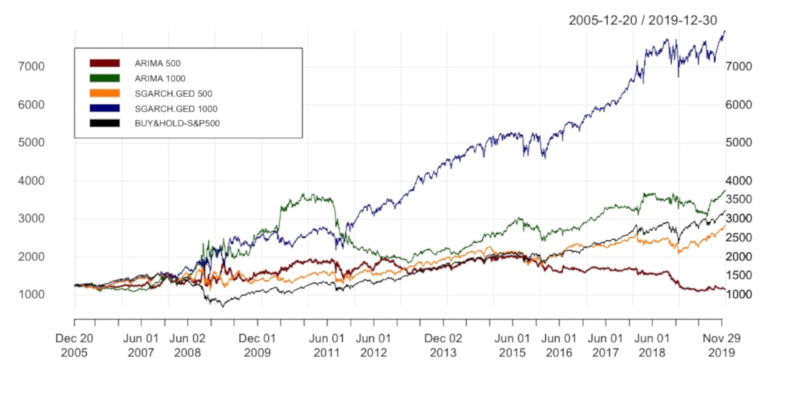
Equity curves of ARIMA(p,1,q) and hybrid models with window sizes = 500 and 1000. In order to simplify the structure of the legend, SGARCH.GED 500/SGARCH.GED 1000 is understood as ARIMA(p,1,q)-SGARCH(1,1) with GED distribution and window size(s) equal to 500/1000 days.

**Figure 10:**
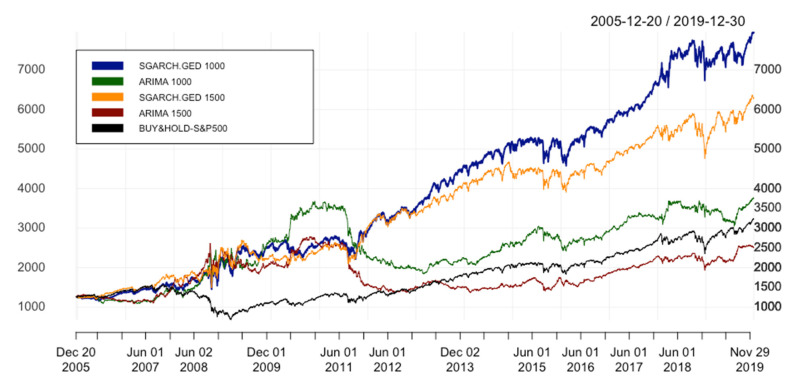
Equity curves of ARIMA(p,1,q) and hybrid models with window sizes = 1000 and 1500. In order to simplify the structure of the legend, SGARCH.GED 1000/SGARCH.GED 1500 is understood as ARIMA(p,1,q)-SGARCH(1,1) with GED distribution and window size(s) equal to 1000/1500 days.

Figure 9 and Figure 10 illustrate the equity curves of tested strategies and the benchmark with the window sizes of 500 and 1500, respectively. Noticeably, in Figure 9, both ARIMA 500 and SGARCH.GED 500 underperform benchmark at the end. We could see that the Buy&Hold strategy is not necessarily the worst for various values. In general, our hybrid models seem to be sensitive to the values of window size. However, we can still conclude that hybrid models outperform ARIMA regardless of the values of window size as an input parameter. To recap, in response to the third research question in this paper, the results obtained from the main test are robust to varying window sizes.

**Figure 11:**
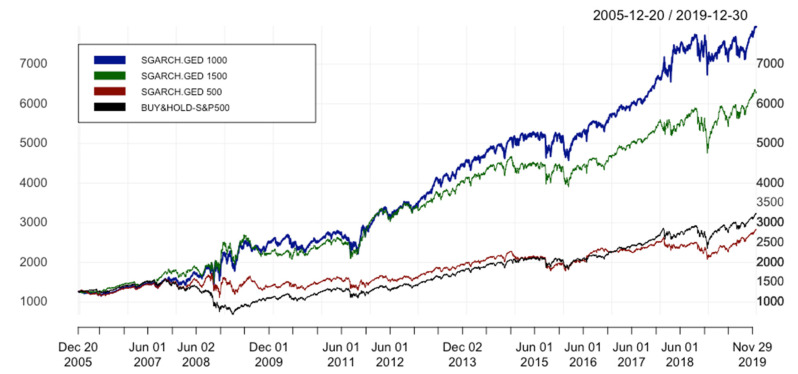
Equity curves of ARIMA(p,1,q)-SGARCH(1,1) with different window sizes. In order to simplify the structure of the legend, SGARCH.GED 500/SGARCH.GED 1000/SGARCH.GED 1500 is understood as ARIMA(p,1,q)-SGARCH(1,1) with GED distribution and window size(s) equal to 500/1000/1500 days.

Table 5 shows that with the highest value of ARC and IR* (14.026% and 0.402, respectively), the hybrid models with the window size of 1000 are the best strategy among these three different window sizes. According to Figure 11, SGARCH.GED 1000 beats all mentioned methods. Based on IR* as the main performance indicator in choosing the best model, SGARCH.GED 1000 outperforms the other hybrid models with different values of s = 500 and = 1500, and their differences are rather significant. In conclusion, the reply to the fourth research question in this paper is the hybrid models are sensitive to different window sizes.

#### 4.2.3. Varying Distributions

Table 6 presents the results of hybrid models with varying distributions, including GED, SNORM, SSTD, and SGED. In terms of error metrics, it is quite evident that ARIMA 1000 has the worst performance with the highest values of MAE, MSE, RMSE, and MAPE in comparison with all hybrid models. Although ARIMA 1000′s KPIs of performance statistics are higher than those of the benchmark, these figures are still lower than all hybrid models. Particularly, in terms of IR*, SGARCH.SNORM 1000 with 0.129, SGARCH.SSTD 1000 with 0.147 and SGARCH.SGED 1000 with 0.119 beat ARIMA 1000 with 0.069.

**Figure 12:**
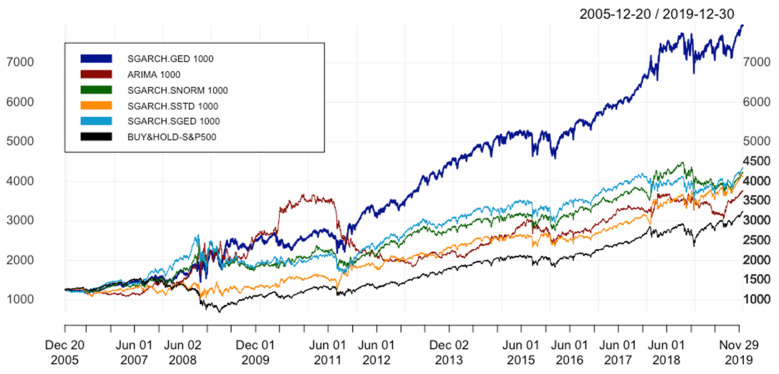
Equity curves of all hybrid models with different distributions. In order to simplify the structure of the legend, SGARCH.GED 1000 is understood as ARIMA(p,1,q)-SGARCH(1,1) with GED distribution and window size(s) equal to 1000 days, similar for SNORM, SSTD, and SGED.

Figure 12 plots the equity curves of all hybrid models with various distributions as well as ARIMA, while window size remains 1000. It can be seen that the cumulative returns of hybrid models with SNORM, SSTD, and SGED distributions show no significant differences at the end, but all of them surpass ARIMA’s. The results in Figure 12 and Table 6 draw the conclusion that regardless of the distributions, our hybrid models are more profitable than ARIMA. In short, the fifth research question can be answered as follows: the results obtained from the main test are robust to varying distributions. As can be seen in Figure 12, it is obvious that the best model is SGARCH.GED 1000, whose ending point is significantly far away from the rest and performance metrics are much better. As for the last research question of this paper, hybrid models are sensitive to different distribution and this with GED distribution outperforms the ones with other distributions, such as SNORM, SSTD, and SGED.

## 5. Conclusions

The main hypothesis of this paper is whether the ARIMA(p,1,q)-SGARCH(1,1) (hybrid model) with window size equal to 1000 can generate an algorithmic trading strategy that outperforms ARIMA(p,1,q). Based on this hypothesis, the research questions are constructed as follows: whether the results in the main test are robust to (RQ1) varying family of GARCH models, (RQ3) varying window sizes, and (RQ5) varying distributions. We also evaluated and examined more research questions regarding whether the performance of hybrid models in the main test change with (RQ2) varying family of GARCH model; (RQ4) varying window sizes and (RQ6) varying distributions.

The dataset used for this research consists of the quotations of the S&P500 index. The data were collected on a daily basis over the period from 1 January 2000 to 31 December 2019. Next, the forecasted value was generated based on the best ARIMA model as a result of the best combination of p from 0 to 5 and q from 0 to 5, which has the lowest value of AIC. A rolling window of 1000 with one day ahead moving was selected for the main test. The vector of forecasted log returns with 3530 elements was generated. Based on these values, we set up the trading signals in which we entered the long position if forecasted log return was positive and the short one if forecasted log return was negative. By assuming the initial investment of $1259.92 (the level of S&P500 index at the starting date), we calculated returns from the starting point of our out-of-sample window on 19 December 2005. Similar steps and process were conducted for ARIMA(p,1,q)-SGARCH(1,1) with GED distribution and window size s = 1000. The difference was that by combining ARIMA with SGARCH to create a hybrid ARIMA-GARCH model, conditional mean and variance could be simultaneously modeled (unlike ARIMA(p,1,q) where only conditional mean is modeled). We then calculated error metrics and performance statistics. Our benchmark was simply the Buy&Hold strategy on the S&P500 index. For the robustness test analysis, we conducted the same procedure step by step, as we did for the main test with changing input parameters, such as replacing GARCH with EGARCH, varying window sizes (500 & 1500), as well as distributions (SNORM, SSTD, SGED). In order to evaluate the performance of these models, we compute error metrics (MAE, MSE, MAPE, RMSE) and performance statistics (ARC, ASD, MD, IR, IR*) and present the equity curve for each model and also the benchmark.

Overall, the result shows that hybrid methods can generate a strategy that can outperform the ARIMA model even when we change our initial assumptions concerning the family of GARCH (RQ1), window sizes (RQ3), and distributions (RQ5). However, they are not always more efficient than our benchmark, as it was in the case of the hybrid model of SGARCH with GED distribution and window size of s = 500. Additionally, the hybrid model of SGARCH with GED distribution and window size equal to 1000 performs the best in comparison to other hybrid models in terms of changing different GARCH model (RQ2), window sizes (RQ4), and distributions (RQ6). Even being introduced as a model which can mitigate the disadvantages of SGARCH, the EGARCH model could not beat SGARCH in this research. To conclude, from the obtained results, the hybrid ARIMA-GARCH can generate a trading strategy that outperforms ARIMA(p,1,q) and should be taken into consideration in predicting returns and building trading strategies instead of applying only ARIMA to the series.

There are some limitations of this paper which can be improved in future works. The first and the biggest is that the proposed ugarchroll function in rugarch package (Author: Alexios Ghalanosonly) supports moving one period ahead only. It is time-consuming and reduces efficiency in practice. This issue may be addressed if we incorporate an extra wrapped function into the main one by making use of the underlying functions in the package. In addition, we applied fixed window sizes of 500, 1000, and 1500 without checking which exact value of the window size would deliver the best results. To solve this issue, we can try to build a loop function with the input parameters that can be a range of window sizes (for example the range of 500, 501, 503, …, 1499, 1500). In this way, the best value of window size which delivers the best trading strategy can be selected. Moreover, we applied a trading strategy based on ideal conditions without any transaction costs, the discussion of the influence of taxes on our results (Batrancea) [42], or a certain threshold. This means we should take into consideration the magnitude of forecast return value instead of building a strategy based on the sign of forecasted values. This issue may be addressed by assigning cost for any transaction and setting a threshold with which we can compare forecasted return value before generating the direction for trading (+1 for entering long or −1 for entering short).

## Figures and Tables

**Table 1:** Descriptive Statistics for S&P500 (Jan 2000–Dec 2019).

Descript Statistics	S&P500 Original Prices	Log Returns
Min	676.5300	−0.0947
1st Quantile	1151.5349	−0.0047
Median	1360.9550	0.0005
Arithmetic Mean	1574.6801	0.0002
3rd Quantile	1986.2225	0.0057
Max	3240.0200	0.1096
Skew	0.9886	−0.2295
Kurtosis	−0.0715	8.6448
Standard Error Mean (se)	8.2559	0.0002
Standard Deviation (sd)	585.5315	0.0119

Note: The table covers the period between 1 January 2000 and 31 December 2019.

**Table 2:** Sets of input parameters (ARIMA/hybrid ARIMA-xGARCH).

Parameters	Values
**Sample sizes**	s ∈ {500, **1000**, 1500} (days)
**Distribution**	**Generalized Error Distribution (GED)** Skewed Normal Distribution (SNORM) Skewed Generalized Error Distribution (SGED) Skewed Student t Distribution (SSTD)
**xGARCH MODEL**	x ∈ {**SGARCH**, eGARCH} (x represents the type of tested GARCH model. In other words, x is either **symmetric** (s)GARCH or exponential (e)GARCH.

Note: The letters in bold represent the parameters in the main test.

**Table 3:** Forecasting performance of ARIMA(p,1,q) and ARIMA(p,1,q)-SGARCH(1,1).

Error Metrics					Performance Statistics				
METHOD	MAE	MSE	RMSE	MAPE	ARC	ASD	MD	IR	IR*
BUY&HOLD S&P500					6.931%	**18.826%**	56.775%	0.368	0.045
ARIMA 1000	12.122	310.372	17.617	0.00775	8.084%	18.878%	50.007%	0.428	0.069
SGARCH.GED 1000	**11.831**	**303.044**	**17.408**	**0.00754**	**14.026%**	18.893%	**25.885%**	**0.742**	**0.402**

Note: In order to simplify the structure of the table, SGARCH.GED 1000 is understood as ARIMA(p,1,q)-SGARCH(1,1) with GED distribution and window size equal to 1000 days. MAE: mean absolute error; MSE: mean squared error; RMSE: root mean squared error; MAPE: mean absolute percentage error; ARC: annualized return compounded; ASD: annualized standard deviation; MD: maximum drawdown; IR = ARC/ASD: information ratio; IR* = (ARC^2 * sign(ARC))/(ASD * MD): adjusted information ratio. Figures in bold indicate the best results.

**Table 4:** Forecasting performance of ARIMA(p,1,q) & ARIMA(p,1,q)-EGARCH(1,1).

	Error Metrics				Performance Statistics				
METHOD	MAE	MSE	RMSE	MAPE	ARC	ASD	MD	IR	IR*
BUY & HOLD S&P500					6.931%	**18.826%**	56.775%	0.368	0.045
ARIMA 1000	12.122	310.372	17.617	0.00775	8.084%	18.878%	50.007%	0.428	0.069
SGARCH.GED 1000	11.831	303.044	17.408	0.00754	**14.026%**	18.893%	**25.885%**	**0.742**	**0.402**
EGARCH.GED 1000	**11.828**	**301.74**5	**17.371**	**0.00753**	11.010%	18.901%	29.150%	0.582	0.220

Note: In order to simplify the structure of the table, EGARCH.GED 1000 is understood as ARIMA(p,1,q)-EGARCH(1,1) with GED distribution and window size equal to 1000 days. MAE: mean absolute error; MSE: mean squared error; RMSE: root mean squared error; MAPE: mean absolute percentage error; ARC: annualized return compounded; ASD: annualized standard deviation; MD: maximum drawdown; IR = ARC/ASD: information ratio; IR* = (ARC^2 * sign(ARC))/(ASD * MD): adjusted information ratio. The figures in bold indicate the best results.

**Table 5:** Performance of ARIMA(p,1,q) and hybrid models in different window sizes.

	Error Metrics				Performance Statistics				
METHOD	MAE	MSE	RMSE	MAPE	ARC	ASD	MD	IR	IR*
BUY & HOLD S&P500					6.931%	**18.826**%	56.775%	0.368	0.045
ARIMA 500	12.216	318.342	17.842	0.00777	−0.573%	18.830%	46.471%	−0.030	0.000
SGARCH.GED 500	11.91	307.812	17.545	0.00758	5.912%	18.871%	35.666%	0.313	0.052
ARIMA 1000	12.122	310.372	17.617	0.00775	8.084%	18.878%	50.007%	0.428	0.069
SGARCH.GED 1000	11.831	**303.044**	**17.408**	**0.00753**	**14.026%**	18.893%	**25.885%**	**0.742**	**0.402**
ARIMA 1500	12.069	308.983	17.578	0.00771	5.005%	18.852%	50.733%	0.265	0.026
SGARCH.GED 1500	**11.825**	303.298	17.415	0.00753	12.186%	18.896%	25.885%	0.645	0.304

Note: In order to simplify the structure of the table, SGARCH.GED 500 is understood as ARIMA(p,1,q)-SGARCH(1,1) with GED distribution and window size s = 500 days, similar for s = 1000 and = 1500 days. MAE: mean absolute error; MSE: mean squared error; RMSE: root mean squared error; MAPE: mean absolute percentage error; ARC: annualized return compounded; ASD: annualized standard deviation; MD: maximum drawdown; IR = ARC/ASD: information ratio; IR* = (ARC^2 * sign(ARC))/(ASD * MD): adjusted information ratio. The figures in bold indicate the best results.

**Table 6:** Performance of ARIMA(p,1,q) and hybrid models in different distributions.

	Error Metrics				Performance Statistics				
METHOD	MAE	MSE	RMSE	MAPE	ARC	ASD	MD	IR	IR*
BUY & HOLD S&P500					6.931%	**18.826%**	56.775%	0.368	0.045
ARIMA 1000	12.122	310.372	17.617	0.00775	8.084%	18.879%	50.007%	0.428	0.069
SGARCH.GED 1000	**11.831**	**303.044**	**17.408**	**0.00754**	**14.026%**	18.893%	**25.885%**	**0.742**	**0.402**
SGARCH.SNORM 1000	11.880	303.151	17.411	0.00758	8.987%	18.890%	33.079%	0.476	0.129
SGARCH.SSTD 1000	11.928	305.642	17.483	0.00762	8.860%	18.881%	28.373%	0.469	0.147
SGARCH.SGED 1000	11.848	302.362	17.389	0.00755	9.201%	18.859%	37.566%	0.488	0.119

Note: In order to simplify the structure of the table, SGARCH.GED 1000 is understood as ARIMA(p,1,q)-SGARCH(1,1) with GED distribution and window size s = 1000 days, similar for SNORM, SGED, and SSTD. MAE: mean absolute error; MSE: mean squared error; RMSE: root mean squared error; MAPE: mean absolute percentage error; ARC: annualized return compounded; ASD: annualized standard deviation; MD: maximum drawdown; IR = ARC/ASD: information ratio; IR* = (ARC^2 * sign(ARC))/(ASD * MD): adjusted information ratio. Figures in bold indicate the best results.

## Data Availability

All relevant data are within the paper.
